# Endoscopic Transoral Odontoidectomy for Bullet Extraction: A Case Study

**DOI:** 10.7759/cureus.80421

**Published:** 2025-03-11

**Authors:** Sebastian Rescalvo Garduño, Juan Manuel Salgado Camacho, Gervith Reyes Soto, Carlos Castillo-Rangel, Alexey N Shkarubo, Ramiro Lopez-Elizalde, Andreina Rosario Rosario, Manuel De Jesus Encarnacion Ramirez

**Affiliations:** 1 Neurosurgery, Regional High Specialty Hospital of Ixtapaluca (HRAEI), Mexico City, MEX; 2 Neurosurgical Oncology, Mexico National Cancer Institute, Tlalpan, MEX; 3 Neurosurgery, ISSSTE Regional Hospital October 1, Mexico City, MEX; 4 Neurooncology, N. N. Burdenko National Medical Research Center of Neurosurgery, Federal State Autonomous Institution, Ministry of Health of the Russian Federation, Moscow, RUS; 5 Oncology, Hospital Civil Dr. Juan I. Menchaca, Universidad De Guadalajara, Guadalajara, MEX; 6 Medicine, Autonomous University of Santo Domingo (UASD), Santo Domingo, DOM; 7 Neurosurgery, Peoples' Friendship University of Russia, Moscow, RUS; 8 Human Anatomy and Histology, N.V. Sklifosovskiy Institute of Clinical Medicine, Moscow, RUS

**Keywords:** craniovertebral junction, endoscopic, endoscopic transoral, extraction, odontoidectomy

## Abstract

This report presents a case of craniovertebral junction (CVJ) trauma caused by a gunshot injury, managed successfully with endoscopic transoral odontoidectomy. The procedure achieved ventral decompression, bullet extraction, and neurological improvement while addressing challenges like severe edema, fragmented dental structures, and cerebrospinal fluid (CSF) leaks using advanced tools and expert planning. A patient with a gunshot wound to the CVJ underwent endoscopic transoral odontoidectomy. Preoperative imaging revealed a bullet near vital structures, prompting surgery for decompression and removal. The patient was positioned with neuronavigation guidance, and a tracheostomy ensured airway management. Odontoid resection and bullet extraction were performed, and a CSF leak was repaired using Duragen and fibrin sealant. The surgery achieved complete bullet removal and cervicomedullary decompression without major complications. Imaging confirmed no residual fragments. Neurological improvement was observed postoperatively, though secondary stabilization was planned due to instability from odontoid resection. Complications, including a CSF leak and soft palate edema, were managed effectively, and the patient experienced progressive functional recovery. This case highlights the endoscopic transoral approach as an effective, minimally invasive technique for managing complex CVJ trauma. Advanced technology and expert planning ensured a successful outcome, emphasizing the need for tailored strategies and further research to validate its broader application.

## Introduction

The incidence of gunshot wounds to the spine accounts for 13-17% of all spine injuries [1]. However, cervical lesions are rarely seen due to the life-threatening conditions of such patients. The incidence of neurovascular lesions is high, and those who survive may become severely disabled. The inherent nature of a gunshot wound is generally associated with stable lesions, and surgical stabilization is rarely necessary [1,2].

Odontoidectomy, which involves removing the odontoid process of the C2 vertebra, is typically indicated for cases of significant anterior compression that cannot be resolved with posterior approaches. It is especially suitable for patients with persistent neurological symptoms after posterior surgeries or those with irreducible anterior compression [2].

The anterior approach to the craniovertebral junction (CVJ) offers direct access to the ventral midline, allowing effective decompression of the lower brainstem and upper cervical spinal cord. This method is critical for conditions where the odontoid process exerts pathological pressure on neural structures. Surgical exposure usually ranges from the inferior third of the clivus to the upper C3 vertebra, with transoral exposure extendable superiorly through transpalatal or transmaxillary approaches if required [3].

Odontoidectomy can be performed via two primary techniques: the transoral and endoscopic endonasal approaches. The transoral approach, first described by Kanavel in 1917, has significantly advanced with microsurgical innovations. Alternatively, the endoscopic endonasal approach, introduced by Kassam et al. in 2005, offers a modern, viable option for accessing this intricate anatomical area [4].

The transoral approach remains a key technique for treating irreducible extradural midline lesions compressing the cervicomedullary junction. Despite its efficacy, risks such as infections, dural tears, and vertebral artery injuries are associated with the method. Additionally, complications from retractor use, including tongue and dental trauma, though less common, must be considered. Significant mucosal bleeding during the procedure may necessitate aggressive measures [5].

However, the transoral approach has inherent limitations. Reaching the C2-3 disc is challenging, and performing a complete C2 corpectomy often exceeds the operative field. Ventral reconstruction using plates or cages is generally impractical, and direct dural repairs in cases of tears are complex. Furthermore, anatomical structures like the Eustachian tube hinder lateral resections. These constraints highlight the importance of thorough anatomical knowledge and careful patient selection [6].

This report presents a case involving a gunshot wound to the upper cervical spine that required a tailored surgical strategy. The injuries included clivus and odontoid fractures, with the bullet trajectory traversing the maxilla, septum, and C1 arch. Due to these anatomical complexities, the endonasal route was unsuitable, making the transoral endoscopic approach the preferred option [7].

Endoscopic transoral odontoidectomy is an advanced technique combining direct access with the advantages of minimally invasive endoscopic technology. It enhances visualization, magnification, and maneuverability, reducing surgical morbidity compared to open approaches. Nevertheless, it requires specialized skills and equipment [8].

This report details the surgical management and outcomes of a patient who underwent endoscopic transoral odontoidectomy for bullet removal. It highlights the technical challenges, anatomical considerations, and postoperative factors inherent in such cases. Additionally, a literature review provides broader context and implications for this method [9].

Advances like neuronavigation and endoscopy have greatly improved the precision and safety of CVJ surgeries. However, these innovations demand significant expertise and awareness of potential complications. By sharing this case and reviewing relevant studies, we aim to enhance collective knowledge and provide valuable insights for neurosurgeons managing similar cases [10].

## Case presentation

This report describes the management of a patient with a gunshot wound to the CVJ, treated using an endoscopic transoral odontoidectomy. The data for this case were collected retrospectively from the patient’s clinical records at the Regional High Specialty Hospital of Ixtapaluca (HRAEI), in accordance with institutional protocols for case documentation.

Case description

A 38-year-old Mexican male patient arrived at the emergency department of the Regional High Specialty Hospital of Ixtapaluca (HRAEI) with a gunshot injury to the cervical spine and exhibited significant neurological decline. On admission, the patient had a Glasgow Coma Scale (GCS) score of 11, which later dropped to 8, necessitating advanced airway management.

**Figure 1:**
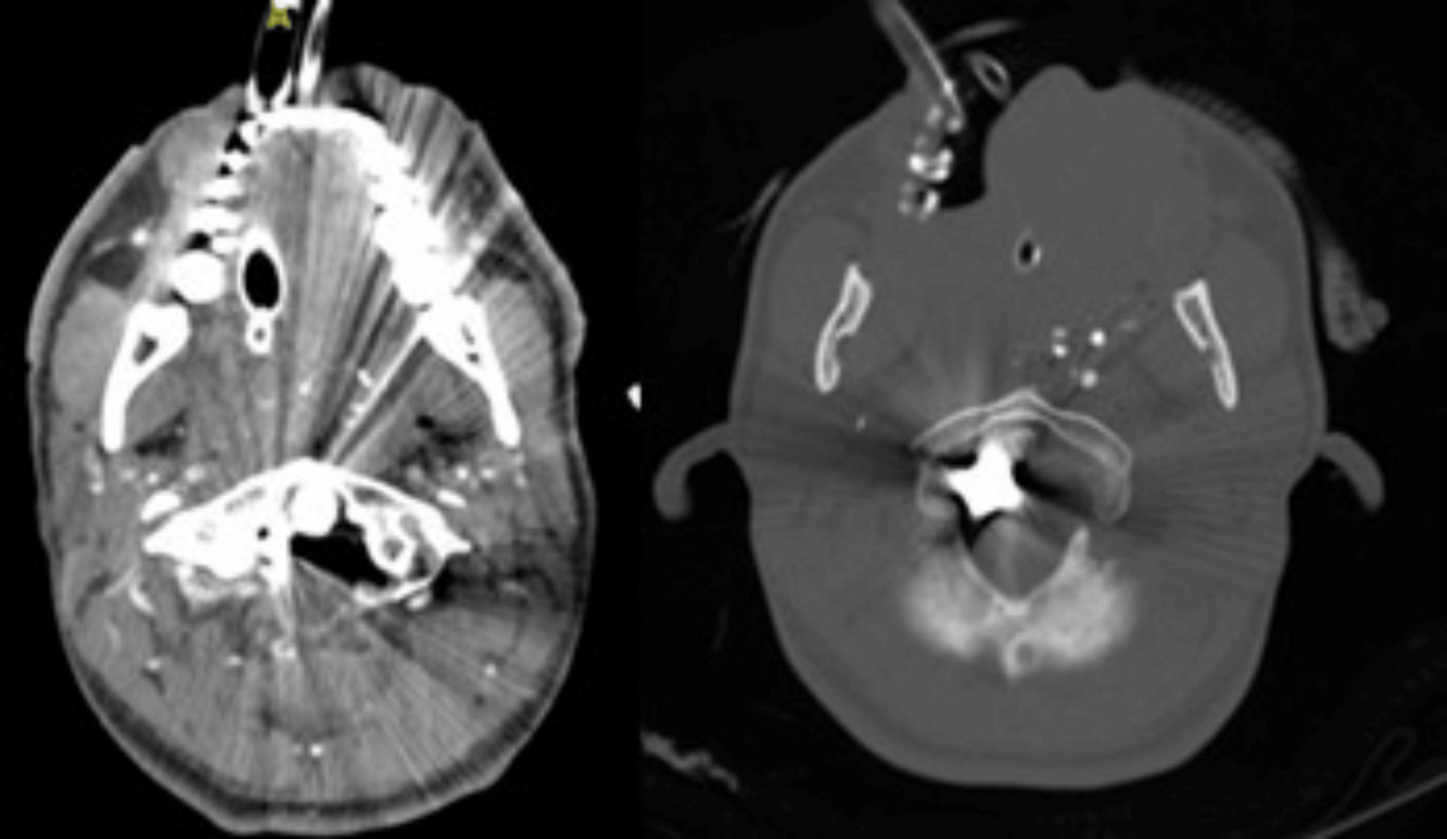
An axial CT scan showed a hyperdense metallic foreign body (bullet) lodged near the lateral aspect of the odontoid process (C2), between the lateral mass of C1 and the odontoid. The image reveals comminuted fractures of the anterior arch of C1, with partial disruption of the cortical structure of the odontoid process. There is significant narrowing of the spinal canal due to the retropulsion of bone fragments, resulting in ventral compression of the cervicomedullary junction. Metallic artifacts from the bullet obscure finer details of adjacent structures, but surrounding fractures and soft tissue damage are clearly delineated. CT, computed tomography

Preoperative non-contrast computed tomography (CT) scans revealed a complex bullet trajectory causing fractures of the clivus, potential involvement of C1, and suspected damage to the odontoid process (Figure 1). These findings indicated the need for surgical intervention to relieve ventral compression and extract the bullet.

Surgical approach

Preparation and Positioning

**Figure 2:**
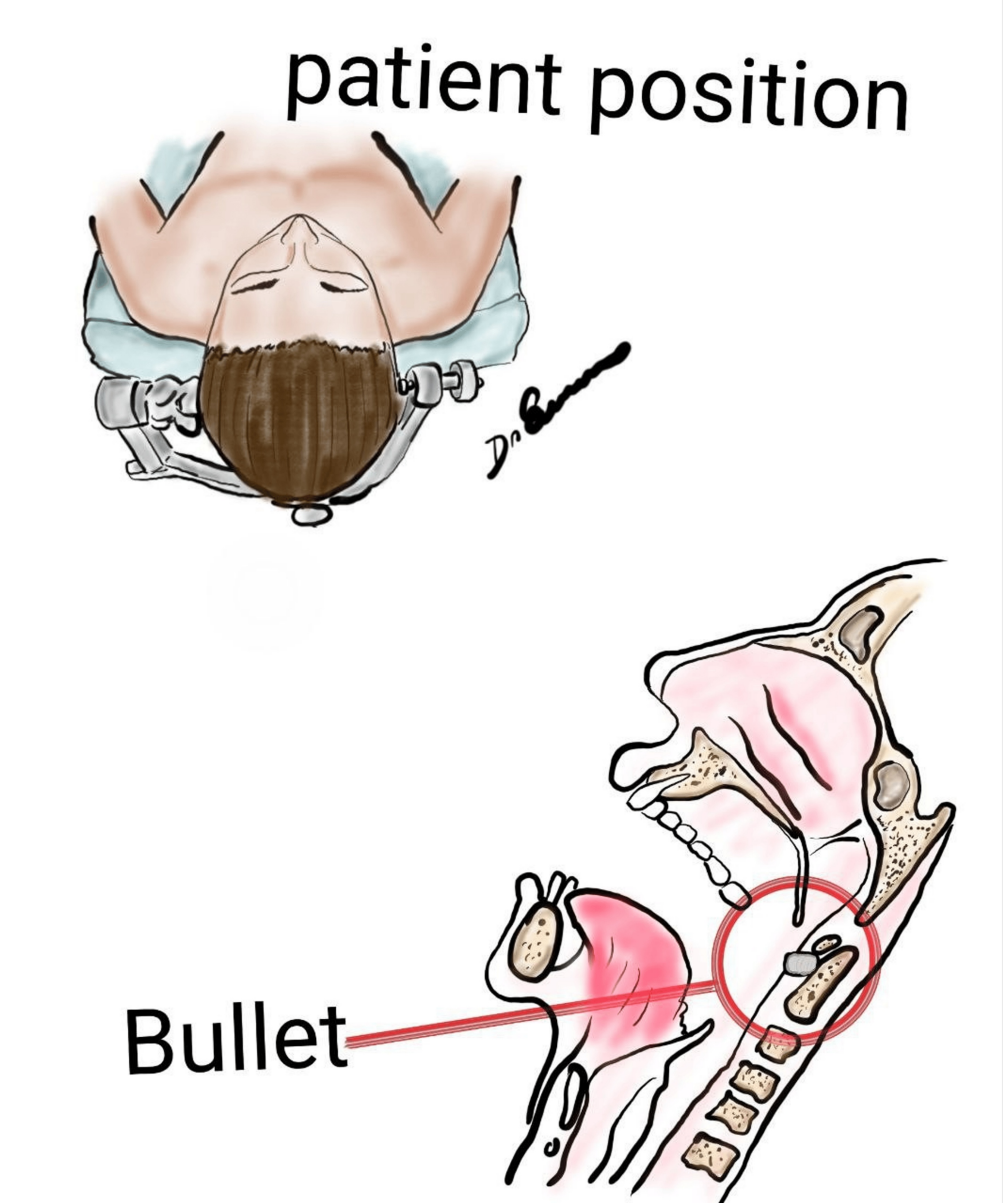
Original schematic illustration showing the patient's position and transoral route to the odontoid. Image Credit: Manuel De Jesus Encarnacion Ramirez

The patient was placed under general intravenous anesthesia. Antisepsis of the cervical and oral regions was performed using iodine-based solutions. To secure the airway, a tracheostomy was conducted. The head was stabilized using a Mayfield head clamp (Figure 2), and neuronavigation was employed to ensure accuracy during the procedure.

Access to the Surgical Site

An incision was made in the edematous soft palate to access the surgical site. Fragmented dental structures were carefully removed to prepare the surgical corridor and minimize mucosal damage.

Odontoidectomy and Bullet Removal

**Figure 3:**
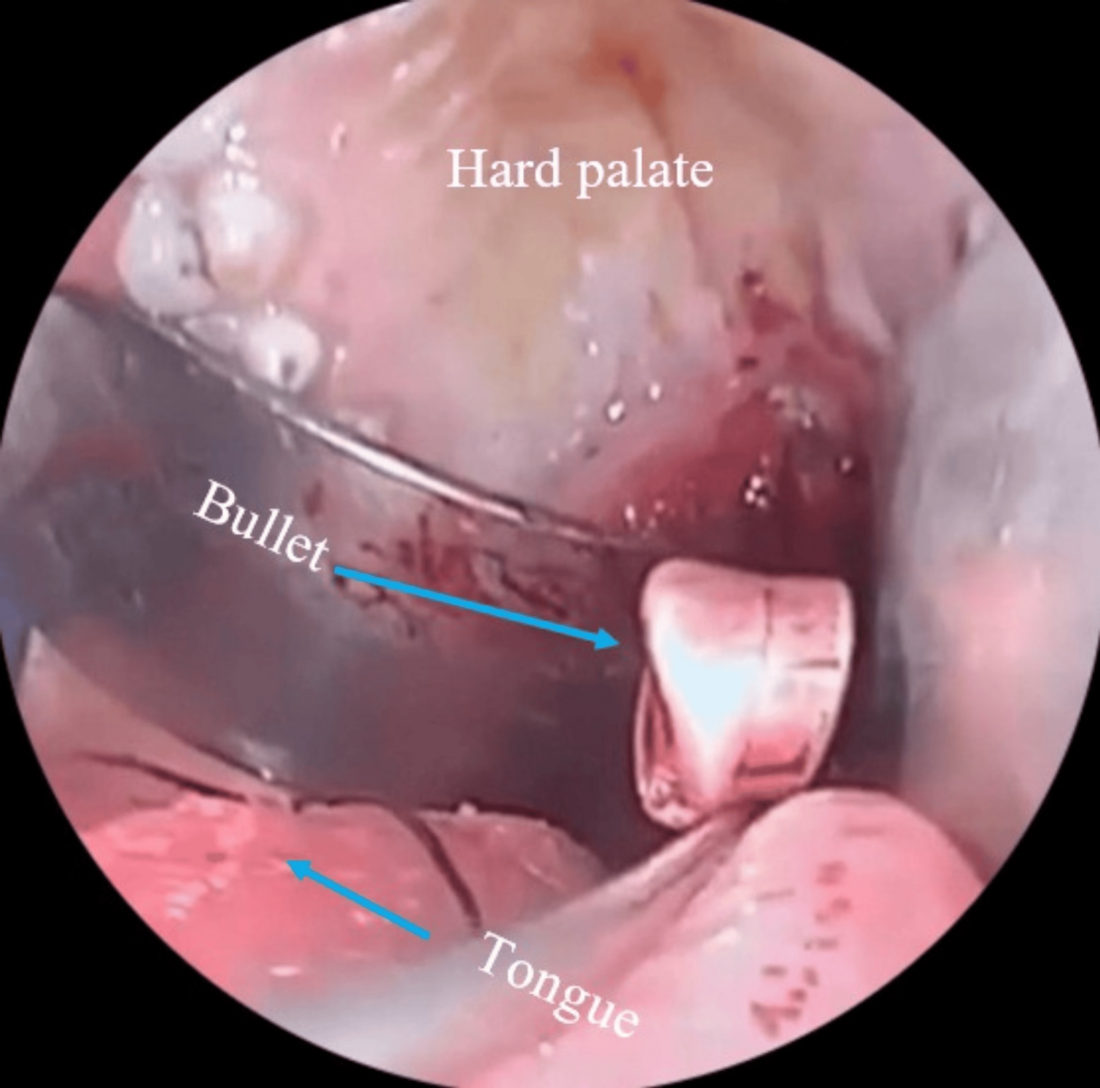
Enhanced visualization and magnification provided by the endoscope showcase the detailed anatomy of the surgical corridor, facilitating safe and effective bullet extraction.

**Figure 4:**
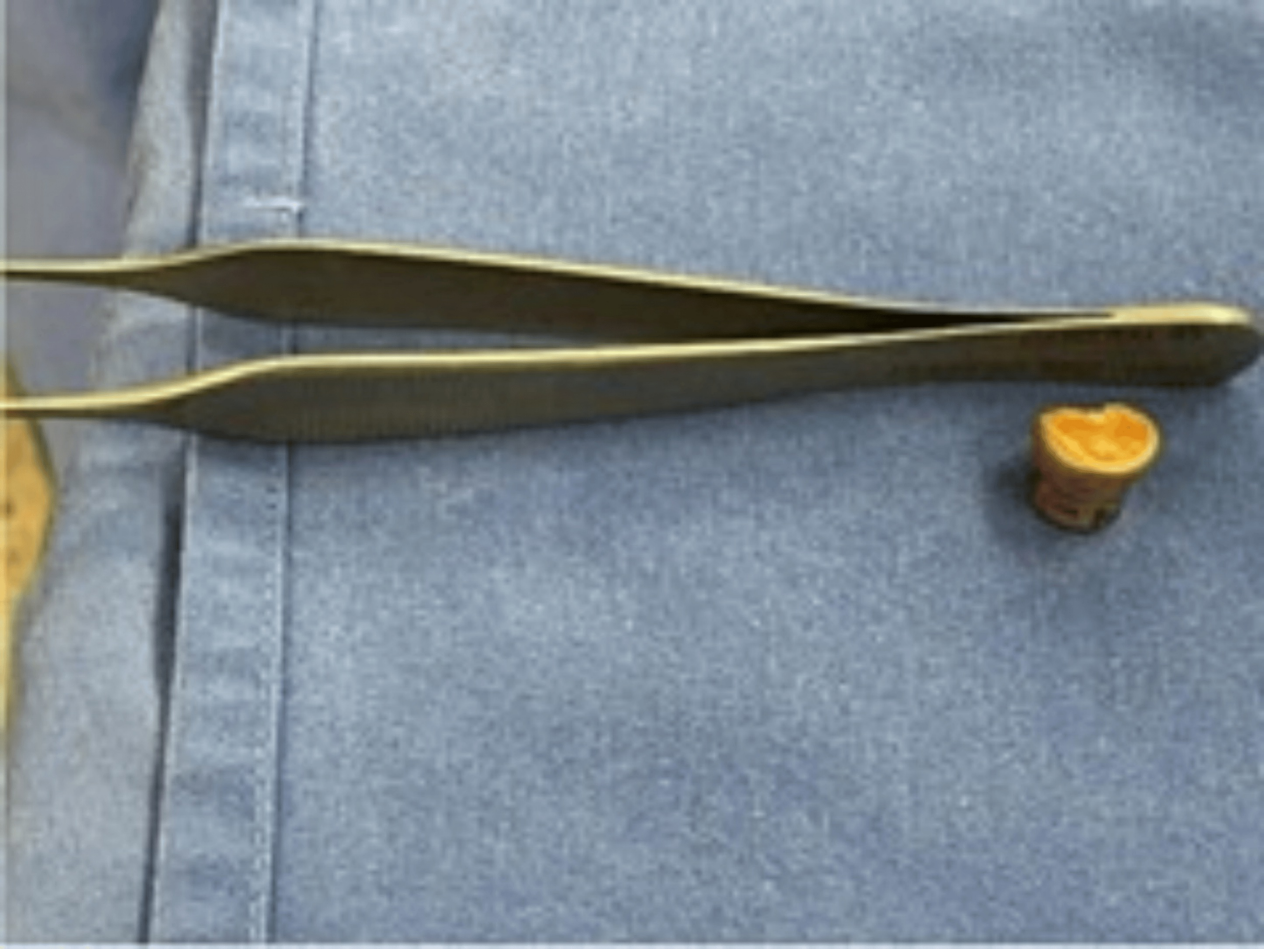
The metallic foreign body is intact, with visible deformation consistent with its trajectory through the CVJ. CVJ, craniovertebral junction

Incremental drilling was performed on the anterior arch of C1 and the odontoid process (dens) of C2, with critical anatomical landmarks, including the vertebral arteries (V3 segment), foramen magnum, and cervicomedullary junction, continuously verified using neuronavigation. The bullet, located between the lateral mass of C1 and the odontoid body of C2, near the vertebral artery groove, was carefully extracted with a pituitary rongeur and handed over to the surgical nurse (Figure 3), maintaining proper chain of custody for forensic purposes (Figure 4).

Management of Dural Tear

During the procedure, a cerebrospinal fluid (CSF) leak was identified and repaired using Duragen and fibrin sealant (Tisseel). Closure of the surgical site was performed in layers, using Vicryl 0 for the muscular plane and Monocryl 2-0 for the mucosa.

Results

Surgical Outcomes

The patient underwent an endoscopic transoral odontoidectomy, which resulted in notable intraoperative findings and outcomes. Severe edema of the soft palate and fragmented dental structures were observed during the procedure. These issues were carefully managed to maintain a clear surgical field and minimize further complications. Incremental drilling and resection of the odontoid process and anterior arch of C1 were performed, successfully relieving compression of the cervicomedullary junction. The bullet, which was lodged laterally to the odontoid body and positioned between the lateral mass and adjacent structures, was successfully extracted and preserved for forensic analysis. During the procedure, a CSF leak was detected and effectively repaired using synthetic dural substitutes and fibrin sealant. Hemostasis was achieved with a total blood loss of approximately 200 cc.

**Figure 5:**
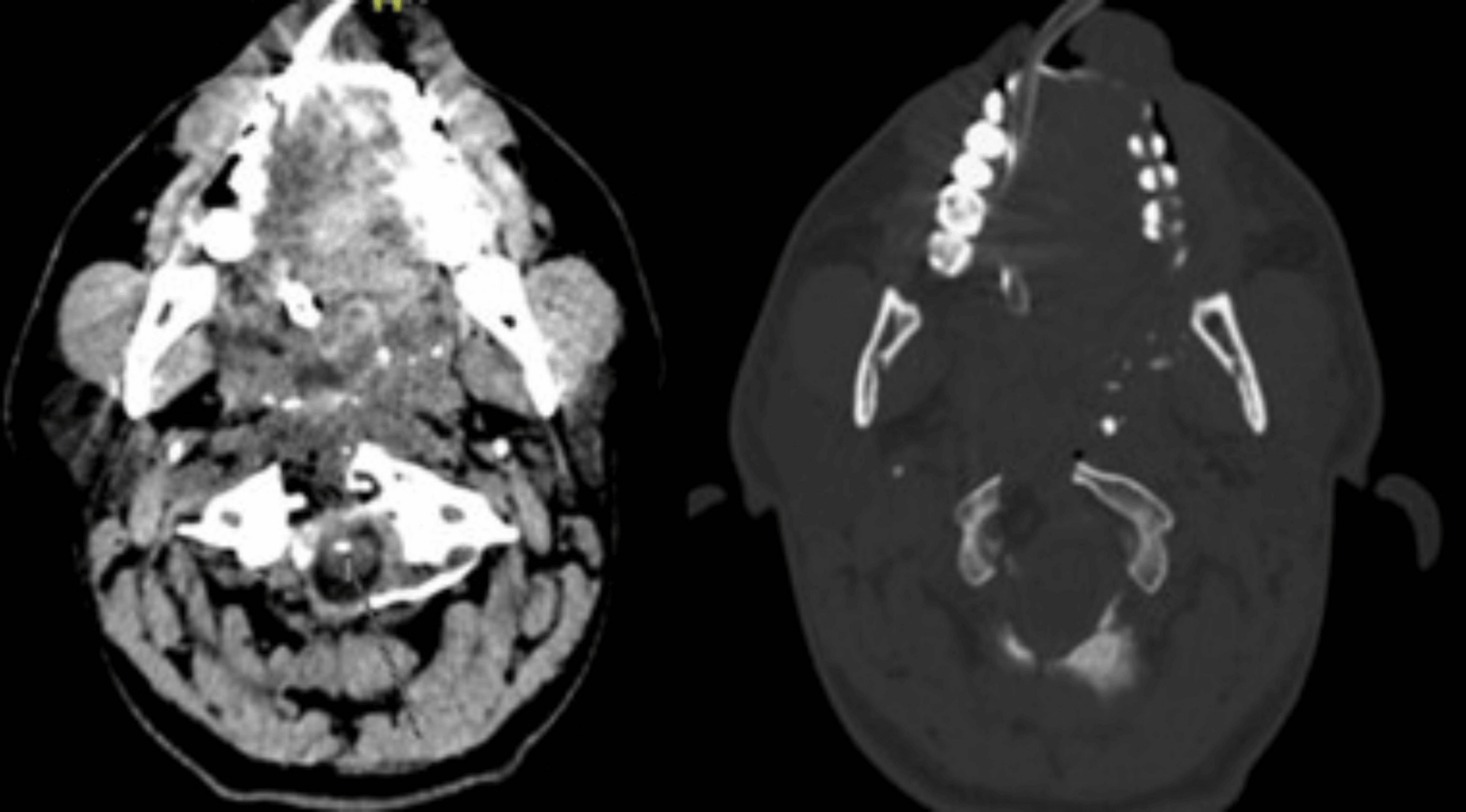
Postoperative axial CT scan demonstrating the successful removal of the hyperdense metallic foreign body (bullet) and decompression of the cervicomedullary junction. The anterior arch of C1 and the odontoid process of C2 show evidence of partial resection, with no residual bone fragments causing significant spinal canal narrowing. Soft tissue edema has resolved, and the surgical field appears clean, with no evidence of hematoma or infection. CT, computed tomography

**Figure 6:**
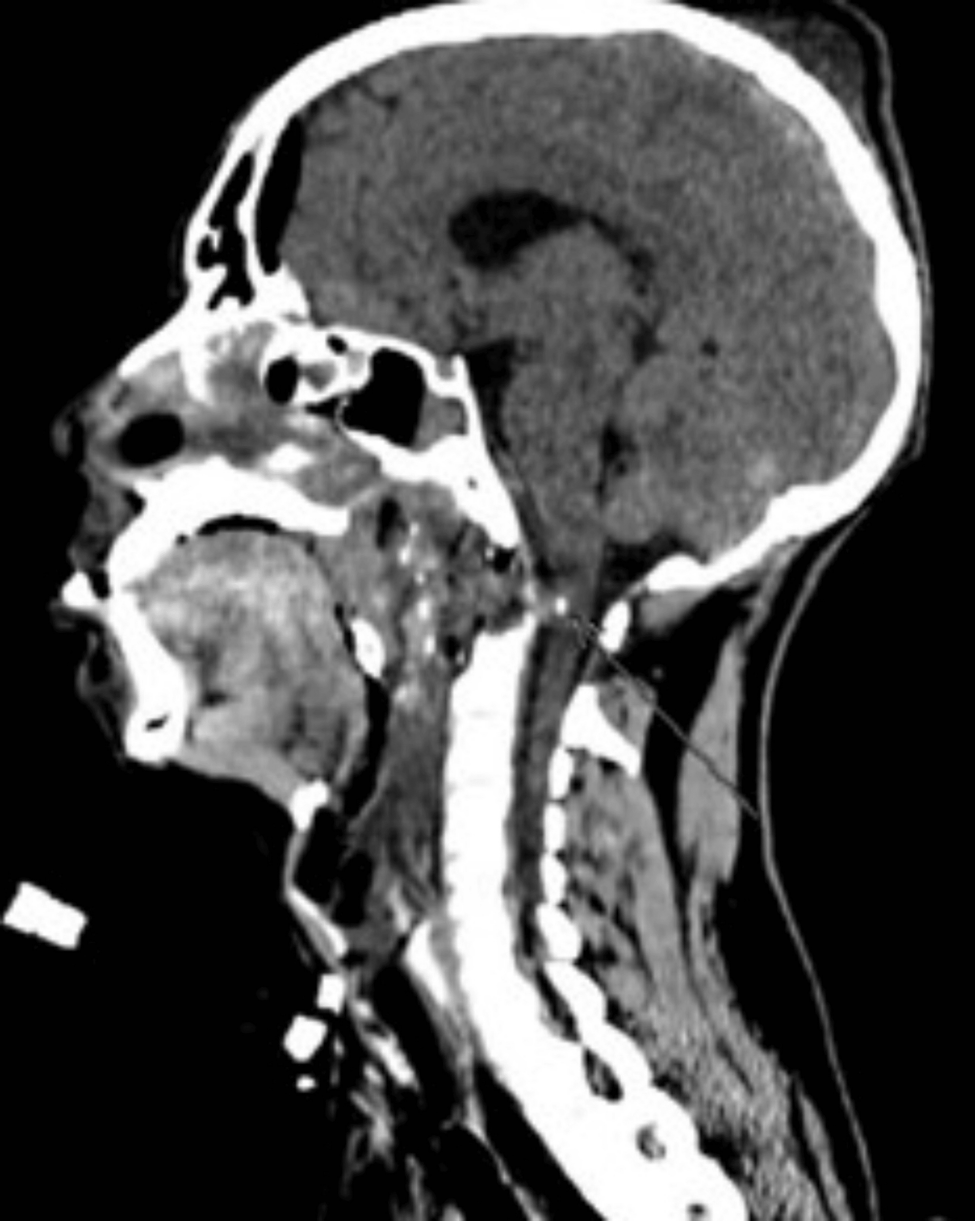
The postoperative sagittal CT scan confirms the absence of the metallic foreign body (bullet), with no residual fragments observed along its trajectory. The odontoid process (C2) has been partially resected. CT, computed tomography

Postoperative imaging confirmed the complete removal of the bullet, adequate decompression of the cervicomedullary junction, and the absence of residual metallic fragments or surgical complications (Figure 5 and Figure 6). The patient demonstrated immediate neurological improvement following the surgery. However, persistent cervical instability, attributed to the partial resection of C1 and the odontoid process, necessitated planning for a second-stage occipitocervical fixation to ensure long-term stabilization.

Complications observed postoperatively included a CSF leak, which was effectively managed during the procedure with no recurrence noted during hospitalization. Mild soft palate edema, a result of the surgery, resolved without additional intervention. Despite elevated preoperative inflammatory markers, there was no evidence of infection at the surgical site or systemically.

Functional Outcome

The patient tolerated the procedure well, showing gradual neurological recovery. Motor and sensory function improved progressively, and the patient was mobilized with a cervical orthosis for temporary stabilization. Discharge planning included close outpatient follow-up and scheduling for definitive stabilization surgery.

## Discussion

This case underscores the challenges of managing CVJ trauma caused by a gunshot wound, particularly when the bullet is lodged near critical neurovascular structures. The use of an endoscopic transoral odontoidectomy proved effective in relieving ventral compression, safely extracting the bullet, and minimizing damage to surrounding tissues.

The procedure successfully achieved complete bullet removal and adequate decompression of the cervicomedullary junction without major complications. A CSF leak detected during surgery was promptly repaired using a collagen matrix graft (DuraGen®: artificial dura mater, Integra LifeSciences, Princeton, NJ, US) and fibrin sealant (Tisseel, Baxter Healthcare Corp., Deerfield, IL), preventing recurrence. Postoperatively, the patient showed significant neurological improvement, with partial recovery of motor and sensory functions. This outcome highlights the advantages of the endoscopic transoral approach, especially in cases where trauma-related anatomical disruptions preclude other techniques, such as the endonasal route.

The surgical process, however, was not without its difficulties. Severe soft palate edema, fragmented dental structures, and multiple fractures of the clivus, nasal septum, and maxilla posed significant challenges. Neuronavigation played a critical role in precisely locating and safely extracting the bullet, underscoring the importance of advanced imaging and modern surgical adjuncts in managing complex CVJ injuries. Despite the success of the decompression and bullet removal, the patient required a second-stage occipitocervical fixation due to instability caused by the partial resection of the anterior arch of C1 and the odontoid process, highlighting a limitation of this approach.

Gunshot wounds to the CVJ are rare and demand highly individualized surgical strategies due to their proximity to vital anatomical structures. The transoral approach, extensively documented in the literature, is a proven method for addressing ventral compression caused by irreducible fractures, tumors, or foreign bodies [11,12]. The endoscopic transoral technique, in particular, offers advantages such as reduced morbidity, superior visualization, and a minimally invasive profile compared to traditional open methods. In this case, the bullet’s trajectory and associated fractures ruled out alternative approaches, such as the endoscopic endonasal route, necessitating a transoral approach. This choice allowed direct decompression of the cervicomedullary junction and bullet extraction, aligning with evidence from prior studies [13,14].

Bayram et al. (2019) reported a rare case of a gunshot wound to the odontoid process that required posterior stabilization, emphasizing the role of individualized imaging and treatment strategies in managing CVJ injuries [15]. While their case involved posterior instrumentation, ours required a transoral approach due to ventral compression and the bullet’s position, demonstrating the variability in surgical decision-making based on anatomical disruption. Similarly, Pattisapu and Al-Mefty (1987) described a gunshot wound to the odontoid process managed conservatively with a halo vest, which was effective in a stable injury without neural compromise [16]. In contrast, the significant neurological deterioration and ventral compression in our patient mandated aggressive surgical intervention. These contrasting cases highlight the importance of tailoring management to the specific clinical presentation [17].

Benzel and Kesterson (1988) also described successful conservative treatment for stable odontoid gunshot injuries with cervical immobilization [18]. However, our case, involving a CSF leak and critical neurological compromise, necessitated surgical intervention to achieve decompression and bullet removal, illustrating the complexity of injuries with CSF involvement [19].

The choice of an endoscopic transoral approach in this case was dictated by the bullet’s trajectory and associated anatomical disruptions. This underscores the importance of comprehensive preoperative planning, multidisciplinary collaboration, and the integration of advanced imaging and modern surgical techniques. Despite the challenges posed by the injury, this approach facilitated a favorable outcome, showcasing its utility in managing complex CVJ trauma [20-22].

Lessons learned

This case provides valuable insights for neurosurgeons managing CVJ injuries caused by gunshot trauma, while also highlighting the limitations of current practices and areas for future development.

Critical Insights for Managing CVJ Gunshot Injuries

Patient-specific planning: CVJ gunshot injuries present unique challenges that require tailored surgical strategies. The bullet’s trajectory, fracture patterns, and the patient’s overall condition must be meticulously evaluated to design an optimal approach.

Multidisciplinary collaboration: Effective management of these complex cases depends on close collaboration among neurosurgeons, radiologists, anesthesiologists, and critical care specialists to ensure the best outcomes.

Preparedness for complications: Common issues such as CSF leaks, infections, and spinal instability necessitate established protocols and resources for prompt intervention.

Advanced technology: Tools like neuronavigation and endoscopy significantly enhance precision and safety, particularly in the intricate anatomy and high-risk environment of the CVJ.

This report has several limitations. First, it is based on a single case, which limits its generalizability. The retrospective design introduces potential biases due to the nature of data collection. Additionally, there is a lack of long-term follow-up, leaving long-term stability and complications, such as infections, unassessed. The absence of a control group means that the efficacy of the transoral approach cannot be fully evaluated. The rarity of CVJ gunshot injuries further limits the applicability of the findings. Furthermore, alternative techniques, such as endonasal or posterior approaches, were not feasible for direct comparison. Ethical considerations, particularly forensic obligations, may have influenced clinical priorities. Neurological recovery was assessed clinically, without the use of advanced tools like EMG, and the technique requires a specific level of expertise, which limits its widespread adoption. There is also significant technological dependence on neuronavigation and endoscopy, which may restrict use in low-resource settings. Finally, the procedure carries risks, including infection, CSF leaks, and tissue damage.

Future directions

Innovations in spinal stabilization may reduce the need for secondary surgeries, while studies comparing transoral and endonasal approaches could refine treatment strategies. Improved surgical tools could help minimize complications, and expanding access to technologies such as neuronavigation and endoscopy may enable wider adoption of these techniques.

## Conclusions

This case report illustrates the successful use of an endoscopic transoral odontoidectomy to remove a bullet lodged at the CVJ following a gunshot injury. The procedure achieved effective decompression of the cervicomedullary junction, removal of the foreign object, and significant neurological improvement, all while avoiding major complications. Challenges such as severe edema, fragmented dental structures, and a CSF leak were addressed with meticulous planning, advanced tools like neuronavigation, and skilled surgical execution, leading to a favorable outcome.

The case underscores the value of the endoscopic transoral approach as a minimally invasive option for managing ventral CVJ trauma, particularly in situations where conventional methods are not feasible due to anatomical or pathological constraints. Its success, however, depends on access to advanced technology, specialized surgical expertise, and careful patient evaluation. The requirement for secondary stabilization procedures highlights the need for a comprehensive treatment strategy and effective multidisciplinary collaboration.
